# Correspondence of the New York Medical Times

**Published:** 1883-03

**Authors:** P. P. Wells


### CORRESPONDENCE OF THE NEW YORK MEDICAL TIMES.

In the February number of the periodical above named are some utterances, which, if not altogether in accord with the truth of the matters there treated, may nevertheless merit a few words by way of comment, lest, being passed in silence, they may be understood as admitted facts. It may not always be well for truth when unjustly or falsely assailed to appear by silence to have confessed judgment against itself. Are the assaults so monstrous, or the spirit which characterizes them so reckless or malicious, or so evidently the outcome of ignorance and stupidity, as to warrant leaving them to refute themselves? is a question which could hardly but arise when reading such statements as those of the New York *Medical Times* which we are about to consider. And it cannot be denied that it is often best to leave such to reap their reward of infamy and discomfiture, which is certain, sooner or later, to come on the falsifier and slanderer as the legitimate fruit of their own doings. At the same time, it is true that there may be circumstances attending the utterances of the reckless which warrant their correction and refutation. These utterances of the *Times* correspondent seem to come to us in such circumstances. With a few of them we now propose to deal.

And first:

"A more momentous question than any hitherto presented is forcibly suggested by the attitude of the Internationals, not a man of whom is in sympathy with the American Institute, nor any of its objects."

In reply to this it would be pertinent to inquire, *How does this writer know this?* And, second, *What are the present "objects" of the*

*Institute?* The recklessness of this statement is quite sufficient for its refutation, if the "*object*" of the Institute remains as it was when this organization was founded. The writer of this paragraph was present and aided with his best endeavors at its birth, and he still lives to testify that the original object of those who with him then engaged in giving existence to this body was to perfect the knowledge of the philosophy of the Homœopathy of Hahnemann's *Organon*, and of the means this employs for healing the sick, and to protect the community from the imposture of those who would, though ignorant, thrust themselves on the public as its competent practitioners. Has the Institute of late abandoned these and taken up some other "*object*"? If the "*object*" of the Institute be a new and a different one, what is it? When answered we may better judge as to sympathies with it. If it be still as it was in the beginning, then we have no hesitation in declaring the alleged want of sympathy with it, on the part of the Internationals, a vile slander. If the Institute has departed from this, its original "*object*," and has, as some of its utterances would seem to indicate, and notably its last resolution at the Indianapolis session, given itself up to efforts to destroy whatever is characteristic of homœopathic philosophy, and to discredit all logical practice under its guidance, then certainly it is more than probable the members of the International Association will, on being interrogated, be found to have "no sympathy" with it. It will be not a little strange if they have. And if this has come to be the "*object*" of the American Institute, the fact is conclusive proof that the International Association was not organized a day too soon. If present experience or memory of more recent utterances in the American Institute suggest that as to the last object of the Institute, the protection of the public from the impositions of incompetent pretenders, is abandoned, it is sad that we are compelled to admit we have no answer to make to the suggestion. The community is not protected, and the profession of Homœopathy is resolved into a sort of a go-as-you-please philosophy and practice. And who is responsible for this? Certainly not the Internationals.

Second:

"They," the Internationals, "hate it," the Institute, "because they are no longer able to manipulate it in their interests. They formed the International Hahnemannian Society with the avowed intention of destroying the American Institute by withdrawing from that body."

Here are two assertions and both are equally false. The writer of this was somewhat in the counsels of those who organized the International Association, at Milwaukee. He never then nor subsequently has heard the first word of a desire to control or "manipulate" the policy of the Institute "in their own interests." They never, not one of those here and so engaged, hinted, by word or act, that he had any interest to subserve other than the interests of Homœopathy as this had been promulgated in the *Organon of Homœopathic Medicine.* No one complained that he had in any way fallen short of any influence in the Institute which was his due. There was complaint that the Institute was not fulfilling the promise of its early existence as to the object of its founders. The complaint, no doubt, was well founded. Neither was there any avowed intention to "destroy the Institute" by withdrawing from that body, or by any other means. It was never proposed in the Association to withdraw. On the contrary, the Association was unanimous in its determination to remain in the Institute, and do it good if possible, and not hurt. The "hate," if there were any, was certainly not with the Association. Is it only with those who thus voluntarily accuse their brethren and assume the position and role of the slanderer?

Third:

"Failing to injure the American Institute by the Milwaukee secession, the Internationals have established an organ and begun a sort of guerilla warfare upon Homœopathy by attacking its 'representative body,' the American Institute."

There never was any secession from the Institute, at Milwaukee or elsewhere, and there was no attempt "to injure" this body by this means or any other. So far from there being a warfare on Homœopathy, as represented in the Institute, by the so-called organ of the Association, it has been the endeavor of that publication to advocate and sustain its philosophy and practice, and not in any wise to oppose the Institute, but only such of its doings and utterances as were subversive of true Homœopathy, and in this we have no hesitation in regarding the "*Organ*" as having done good work. When the Institute would, by its heretical utterances, do its utmost to destroy that for which its organization was created, the "Organ" uttered its rebuke, and it was well it should do so, and it will be well for the Institute and that which it has so greatly misrepresented if the result of this merited rebuke shall be an amendment of its ways and *resolves.* The greatest regret of the Internationals was that by wrong-doing, as they regarded it, the Institute had so greatly injured itself.

Fourth:

"In view of these facts, is it wise to allow the Internationals to remain in the American Institute? Should they not be compelled to abandon the exclusive dogmas of the 'minimum dose of the dynamized drug,' or, failing in this, be read out of the American Institute?"

Wise or unwise, the question of "reading out" would seem to be hardly called for as to those the "*Times*," has already charged with wrong-doing, in that they have of their own volition seceded from the body which, at its behest, is to do the "reading out." In view of these facts, we do not remember a more stupid suggestion than this of "reading out" those who are declared to have taken themselves out of their own volition. But it is perfectly evident the "*reading out*" was not suggested by a "view of these facts" but solely by that *bête noir* of the likes of the *Times*—"the minimum dose of the dynamized drug." How about that final resolution of the Indianapolis session? Was the liberty there declared to be essential to a right doing of all professional duties to do and believe in everything, good and bad, except this hated "minimum dose of the dynamized drug"? This is how a late president of this Institute speaks of this *red rag*, the dose, to all of the likes of our "*Times*:"

"The rule with all homœopaths—the invariable rule—is: the smallest quantity of medicine to accomplish the desired result. If an established cure result from the prescribed agent, no matter how small the dose or how difficult for us to account for its action, that agent deserves and should receive credit for the result; and cures—authentic cures—without number have been made with these small doses." *

This is no doubt true. Will the *Times* please see to it, that when those are "read out" of the Institute because of their faith in this "exclusive dogma" of the "minimum dose," they be cheered with the genial companionship of our excellent late President? And, then, this "dogma" is "exclusive" of what? Of nothing, that we can see, except it be of so much of the drug as is not needed for the cure. It excludes nothing else, and as drugs are only sick-making substances, all of them given not needed for the cure are only adapted to add to the sick condition it is our duty and object to heal.

---

*Dr. Dowling's reply to Professor Palmer, *Old School Medicine and Homœopathy*, (reprint) p. 23.

Is this exclusion so great a crime as to call for the "reading out" from our "great representative body" of those who would thus avoid so great a danger?

Fifth:

"Homœopathy is now seeking national, state, and municipal recognition."

We submit that Homœopathy is seeking no such thing. It may be true that there are those who are ambitious of being regarded as the representatives of this great philosophy, who are seeking such a recognition as will give *them* a kind of importance not otherwise belonging to them, but with this seeking Homœopathy has nothing to do. It accepts and needs as little from this or them as they represent of Homœopathy itself—less than this cannot well be conceived of. Homœopathy seeks no such recognition. It claimed and received long ago the recognition of those in authority, the outcome of which has been the legal incorporation of its colleges, hospitals, dispensaries, State and county societies, and all else needed to place it before the law, and as to all rights, on the same plane as that occupied by any school of medical philosophy and practice, even the most favored. In these matters there is nothing for it to seek. The seeking is all of the selfishness of individuals, not in the least of any need or right of our school.

Sixth:

"It," the Institute, "declared that palliative medicine is the common property of the whole medical profession, and Homœopathy must openly assert its rights therein."

Did it? Then, no doubt, they were guilty of doing a supremely ridiculous thing. Doing this, they show so great ignorance of the nature and evils of palliatives that they are earnestly referred for information on these matters to the *Organon of Homœopathic Medicine*, where the subject is fully treated and in a masterly manner, altogether worthy the attention of these, our so ignorant brethren. They may be able to learn there that so far from these palliatives having any part in homœopathic philosophy or practice, they are wholly outside of these and in their nature antagonistic to the law of similars, and that it has no "rights" in them to "assert openly" or otherwise.

Seventh:

"There was not a dissenting voice on the indorsement of President Breyfogle's address," etc.

This may be just so, but if true, there can be no stronger proof of how far the Institute has fallen from the high standard given to the philosophy and practice of our school by its venerated and enlightened author. So far was this performance from being an "enunciation of homœopathic doctrines," that we are quite sure Hahnemann would not have recognized its utterances as inspired by anything he had taught. And we may add, if the address received, as stated, the unanimous approval of the Institute, it is a sad proof of the ignorance of those present* of that which constitutes true Homœopathy, *i. e.*, of the Homœopathy which Hahnemann taught, and we know of no other.

Eighth:

"It," the Institute, "declared that *similia similibus curantur* is only one of several known laws of cure, available alike to Homœopathy and scientific medicine."

What then? Did the Institute "declare" in what any one or more of these "known laws" consist? Not so. They were only guilty of another supreme folly in this general declaration, which amounts to just so much empty air. There may be other laws of cure than that we profess to accept as our guide. He would be a bold man who would deny this possibility. But it is certain we know no other, and there can be no great risk in affirming, in the absence of their statement of any one of these known laws, neither does the Institute. It can hardly be held as unreasonable if we express the hope, if the Institute ever assembles again, it will have grace enough to refrain from further general utterances of such empty bosh. And then, the expression, "Homœopathy and scientific medicine," is decidedly good. Has the world any "scientific medicine," meaning by this a science of healing the sick, other that of the homœopathic therapeutics? We know no other *scientific* medicine. We do know of many and oft-repeated like pretenses of a something of supposed value presented to the world under this garb of "scientific medicine." Indeed, the expression is so good, it seems to have turned the heads of many of these pretenders. Is this writer in the *Times* one of the victims?

Ninth:

"It," the Institute, "declared that without drug matter there is no drug force," etc.

---

* It should be remembered that the whole of those present and acting at any session are but a minority of the homœopathic profession in the country, and it may be, and often is, even of the membership of the Institute itself.

This is simply another added foolishness. There are unquestionably hundreds of members of this Institute who have seen cures and cures wrought by doses of medicine in which no trace of drug matter could be detected as matter, and yet the patients have responded to the power of the doses as readily and completely as if these had been loaded down by grains, scruples, or drachms. Indeed, if those who have witnessed such results are not mistaken, the cures have been more prompt and perfect than those resulting from the massive doses inculcated by the likes of this writer in the *Times*. It may as well be known that this matter of the curing agent in the drug is one over which neither the Institute nor the New York *Medical Times* has any control. It was fixed before the birth of either, by one who has not yet subjected his works to their revision.

Now, in conclusion, it may be permitted to ask why this hating and hateful wrath which has been poured without stint on this Association, the objective of which was and is, as to the Institute, only to do this body good. And why, of all men, should it have come from those who claim to be friends and advocates of this Institute? Has any other benevolence been met by a similar return in modern time! The writer is witness to the good intentions of the members of the Association as to all of the Institute, which intelligent homœopathists could approve. Indeed, he has no doubt these members, members of the Institute as well, would, if it had been possible, have saved the older organization from its late foolish utterances, some of which have just passed in review before us. Is it that they were not able to prevent these that they have been made objects of so great abuse, and even to the extent of threats of "*reading out*" from the body on which they were willing to expend so great kindness? Not this at all. It is more than probable they were so greatly in the dark as to their needs as never to have realized how great these were, and, therefore, had no just appreciation of this kindness, great and sincere as it was. No. The *fons et origo* of all this wrath and outcry cast at the Association is found, and only found, in the "*minimum dose of the dynamized drug.*" Anything but this has ample protection in that supremely silly "*last resolution,*" but not this. This is not to be tolerated. Those who will not "abandon this exclusive dogma" are to be visited with condign "*reading out.*" Nothing else will allay the excitement of this *red rag*. The Association regard this as an essential integral of homœopathic philosophy. And yet this body who resolve perfect and entire liberty of opinion are advised to "*read out*" such members as have the hardihood to abide by this integral element of law! Have those who so advise considered how this "*reading out*" would look beside that famous, or infamous, *last resolution?* P. P. W.

